# A Dendrite-Autonomous Mechanism for Direction Selectivity in Retinal Starburst Amacrine Cells

**DOI:** 10.1371/journal.pbio.0050185

**Published:** 2007-07-10

**Authors:** Susanne E Hausselt, Thomas Euler, Peter B Detwiler, Winfried Denk

**Affiliations:** 1 Department of Biomedical Optics, Max-Planck Institute for Medical Research, Heidelberg, Germany; 2 Department of Physiology and Biophysics, University of Washington, Seattle, Washington, United States of America; Harvard University, United States of America

## Abstract

Detection of image motion direction begins in the retina, with starburst amacrine cells (SACs) playing a major role. SACs generate larger dendritic Ca^2+^ signals when motion is from their somata towards their dendritic tips than for motion in the opposite direction. To study the mechanisms underlying the computation of direction selectivity (DS) in SAC dendrites, electrical responses to expanding and contracting circular wave visual stimuli were measured via somatic whole-cell recordings and quantified using Fourier analysis. Fundamental and, especially, harmonic frequency components were larger for expanding stimuli. This DS persists in the presence of GABA and glycine receptor antagonists, suggesting that inhibitory network interactions are not essential. The presence of harmonics indicates nonlinearity, which, as the relationship between harmonic amplitudes and holding potential indicates, is likely due to the activation of voltage-gated channels. [Ca^2+^] changes in SAC dendrites evoked by voltage steps and monitored by two-photon microscopy suggest that the distal dendrite is tonically depolarized relative to the soma, due in part to resting currents mediated by tonic glutamatergic synaptic input, and that high-voltage–activated Ca^2+^ channels are active at rest. Supported by compartmental modeling, we conclude that dendritic DS in SACs can be computed by the dendrites themselves, relying on voltage-gated channels and a dendritic voltage gradient, which provides the spatial asymmetry necessary for direction discrimination.

## Author Summary

The visual system dedicates substantial resources to detecting motion and its direction. For more than 40 years, researchers have tried to decipher the underlying computational mechanisms by which retinal neurons compute directed motion. One type of retinal interneuron involved in direction discrimination is the “starburst” amacrine cell. Starburst-cell dendrites are strongly activated by visual motion from their somata towards the dendritic tips, but not by motion in the opposite direction. It has been proposed, for example, that directional selectivity arises from lateral inhibitory interactions in which activated cells inhibit their neighbors. However, despite extensive modeling, the underlying physiological mechanism has remained elusive. Here, by combining whole-cell recordings, two-photon microscopy, and modeling, we show that discrimination of motion direction in starburst-cell dendrites does not require lateral inhibitory interactions in the retina, but can be generated by a “dendrite-autonomous” computation, which relies on intrinsic electrical mechanisms. Blocking inhibitory interactions does not eliminate directional responses, whereas differential activation of voltage-gated membrane conductances and a dendritic voltage gradient can provide the necessary spatial asymmetry to produce directional signals. The computation underlying dendrite-autonomous direction selectivity may represent one of the most intricate examples to date of dendritic information processing.

## Abstract 3

Direction selectivity in starburst amacrine cells in the retina can arise from voltage-gated channels and a dendritic voltage gradient in the absence of lateral inhibition.

## Introduction

**Figure 1:**
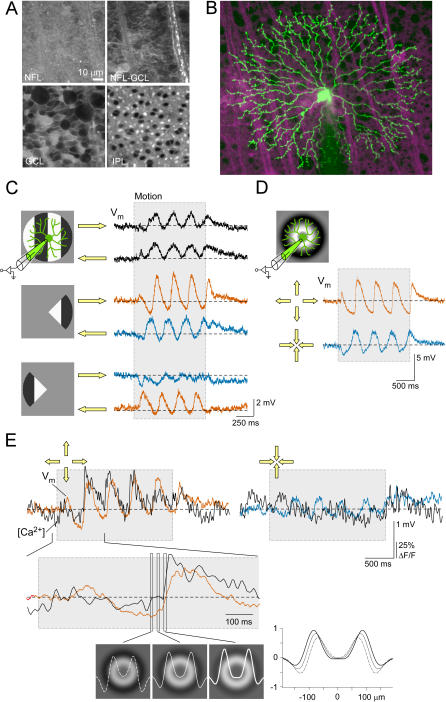
Voltage and Ca^2+^ Responses of Starburst Cells to Moving Gratings and Circular Waves (A) Two-photon micrographs of a living, flat-mounted retina stained with Sulforhodamine 101 (different focal planes: GCL, ganglion cell layer; IPL, inner plexiform layer; NFL, nerve fiber layer). ON (displaced) starburst amacrine somata can be identified by shape and size (center of lower left panel). (B) Cell from (A) filled with the Ca^2+^ indicator dye Oregon-Green 488 BAPTA-1 (green) via the patch electrode (shadow from below; maximum-projected image stack; magenta: sulforhodamine). (C) Voltage responses (*V*_m_) evoked by a bar grating (4 Hz; 72% contrast; period 192 μm) moving left or right (yellow arrows) presented to the full field or “behind” masks (gray) recorded from a different SAC. Left: stimuli and their positions relative to the cell. Right: somatic voltage (gray area indicates motion duration). (D) Voltage responses to circular wave stimulation (2 Hz; 72% contrast; period: 192 μm, see Materials and Methods for details) expanding (CF, orange) or contracting (CP, blue). (E) Simultaneous recording of somatic voltage (orange, blue) and dendritic [Ca^2+^] (black) to circular-wave stimulation. Below: response to CF motion at higher magnification. Gray rectangles indicate duration of stimulus presentation. Radial distribution of normalized stimulus intensity for the three frames are shown as curves on frames (white) and as overlay (plot). (C and D) show averages of three trials, (E) shows single trials; *V*_Rest_ (mV): (C) −43, (D) −61, and (E) −49.

The detection of image motion and the computation of its direction and speed is a major function of the visual system. It is needed for the trajectory prediction of moving objects and provides information about motion relative to the environment [1,2]. In addition to being an intensely studied example of retinal signal processing, motion detection represents an important class of computational problems in neuroscience: the detection of spatiotemporal patterns. The retina's ability to detect the direction of image motion was discovered more than 40 y ago when Barlow and his colleagues [3] found direction-selective (DS) ganglion cells (DSGCs). It was later shown that DSGCs receive DS synaptic input [4–6], which suggested that the direction of motion is computed by retinal interneurons. This was confirmed directly by showing that starburst amacrine cells (SACs; Figure 1) [7,8], which provide input to DSGCs [9–11] and had been proposed early on to participate in the DS computation [12–14], generate DS Ca^2+^ signals in their dendrites [15].

Optical recordings of visual stimulus-evoked Ca^2+^ signals also showed that dendritic sectors can be activated independently and that the strongest [Ca^2+^] increases are in the distal portions of the dendrites, near the output synapses [15]. Electrical isolation of sectors from each other, which is necessary for dendritic processing to be local [14,16,17], is provided by the peculiar dendritic morphology of SACs (Figure 1B) [8,18,19] and a low impedance at the soma [20]. In addition, each sector, unlike the whole cell, is functionally polarized in that synaptic inputs from bipolar and amacrine cells are distributed along the entire dendrite, whereas synaptic outputs are restricted to the distal part [21].

At least two fundamentally different mechanisms have been proposed for dendritic DS in SACs: dendrite-intrinsic electrotonics (e.g., [22,23]) and lateral inhibition (e.g., [14,24]). Despite extensive modeling (reviewed by [25,26]), the physiological mechanism underlying intrinsic DS computation in SAC dendrites has remained elusive. In passive models [22,23], the differences that are found between the voltages induced by centrifugal (CF, towards the dendritic tips) and centripetal (CP, towards the soma) stimuli are small and too sensitive to stimulation parameters to explain the robust direction discrimination seen in DSGCs over wide contrast [27] and velocity ranges [28]. Passive models also predict smaller voltage responses at the soma for CF than CP stimuli [23,29], whereas experiments show the opposite [15,30–32]. The inability of passive models to account for these properties of SAC DS suggests that active (voltage-gated) conductances or strong interactions between inhibitory and excitatory synaptic inputs [14,33] are involved.

To investigate this further, we focused on the electrical properties of SACs using whole-cell recording, two-photon imaging, and compartmental modeling. We show that DS in SACs can be generated by a dendrite-autonomous mechanism that relies on active conductances and a somatodendritic voltage gradient.

## Results

### Direction-Dependent Voltage and Ca^2+^ Responses

In axon-bearing neurons, the somatic voltage is always a direct measure of the cell's output signal, i.e., action-potential generation. This is not the case in SACs, in which synaptic output depends on the voltages in the distal dendritic branches, yet the soma is the only place SACs can be recorded electrically. Nonetheless, even weak electrotonic coupling of the dendritic sectors to the soma can provide information about electrical events in one or more dendritic sectors, but only as long as these signals reinforce rather than interfere with each other. In fact, somatic voltage responses are not systematically DS when probed with full-field moving grating or bar stimuli (Figure 1C, upper traces, and [15,30]), probably because signals from different branches arrive at the soma with different, even opposite, phases and thus cancel. This is not surprising since their circular symmetry alone precludes a DS response of SACs to full-field stimuli.

Cancellation can be avoided either by restricting stimulation to part of the dendrite (Figure 1C; middle and lower traces) or by ensuring—with circularly symmetrical stimuli—that all branches respond in the same way. Then signals from different branches are in phase and reinforce at the soma. Here, we used CF and CP circular wave stimuli (drawing in Figure 1D, see Materials and Methods and [15]), which evoke distinctly DS somatic voltage (Figure 1D) and dendritic Ca^2+^ responses (Figure 1E, see also [15]).

Dendritic Ca^2+^ responses (Figure 1E) to light stimuli were seen in a minority of cells (nine out of 50) while the patch electrode was attached, whereas electrical responses were present in all cells. In some cells (eight of 16) that remained intact after the patch electrode had been retracted, light-evoked Ca^2+^ responses developed after about 15 min. When present, dendritic Ca^2+^ responses (Figure 1E) were similar to those observed in cells loaded via high-resistance microelectrodes [15]. In simultaneous optical and electrical recordings, we found that [Ca^2+^] and voltage rise sharply and in parallel as the bright phase of the circular wave enters the distal dendritic field (Figure 1E). This suggests a common physiological cause, possibly the opening of voltage-gated Ca^2+^ channels (VGCCs).

### Motion Direction–Dependent Nonlinearities

**Figure 2:**
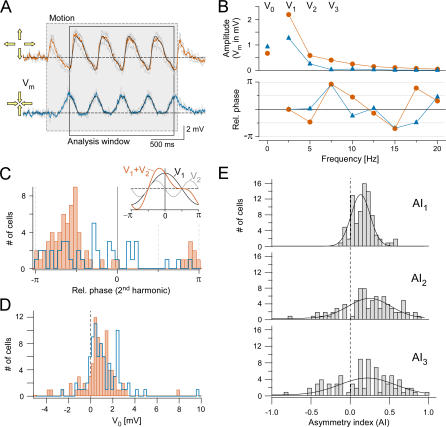
Quantification by Fourier Decomposition (A) Voltage responses evoked by circular wave stimuli; gray: individual trials, orange and blue: averages for CF and CP motion, respectively; black: reconstructed waveforms; frame: trace segment used for analysis (*V*_Rest_ = −60 mV). Gray area indicates motion duration. (B) Amplitudes (top) and relative phases (*φ*_2_ − 2 · *φ*_1_, bottom) versus frequency *(f )* for the averaged responses in (A). Symbols (*V*_0_ at *f* = 0; *V*_1_ at *f*_1_; *V*_2_, *V*_3_... at 2·*f*_1_, 3·*f*_1_,...) are measured points; connecting lines are only a guide for the eye. Note that the phases for the CF motion decrease linearly except when wrapping around (at −π, π). Reconstructions of the waveforms using *V*_0–3_ and their phases shown as black traces in (A). (C) Histogram of the relative (rel.) phase shift (*φ*_2_ − 2 · *φ*_1_) between fundamental and second harmonic (orange for CF, *n* = 65; blue for CP motion, *n* = 41 cells; only cells with V_2 _≥ 0.3 mV included; stimulus: 2.5–3 Hz; 46%–73% contrast; period: 192 μm). Inset: fundamental and second harmonic generate a steeply rising flank when the relative phase is close to −π/2. (D) Distribution of *V*_0_ (*n* = 83, same cells as in [C]) for CF (orange) and CP motion (blue). (E) Distribution of asymmetry indices, *AI*_1_, *AI*_2_, and *AI*_3_ (*n* = 83 cells; all *AI*s are significantly different from zero with p_AI1,AI2,AI3_ ≤ 0.0001).

Nonsinusoidal temporal responses (Figure 2A), particularly to CF motion, indicate the presence of a nonlinearity, because a linear system never generates any “new” frequencies and sinusoidal circular-wave stimuli contain no temporal frequencies other than the fundamental. A sensitive way to recognize and quantify nonlinearity in responses evoked by sinusoidal stimuli is Fourier analysis. The response waveforms can often be well approximated by a sum of a direct current (DC) voltage component (*V*_0_), a voltage component at the fundamental frequency (*V*_1_), and a few low-order (second and third) harmonic voltage components (*V*_2_ and *V*_3_) (Figure 2A and 2B; see also inset in Figure 2C). Component amplitudes were used to quantify the nonlinearity (Table 1) [34].

**Table 1:**
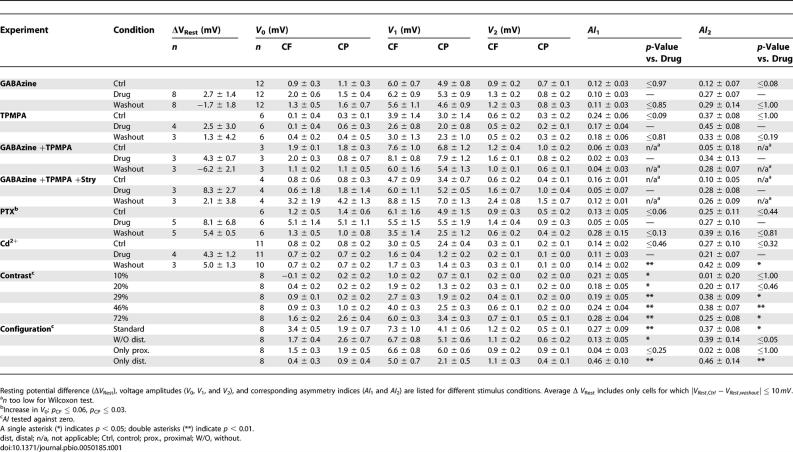
Summary of Results

Circular wave stimuli generated responses with harmonics that were larger for CF than CP stimuli (Figure 2B); this was most evident for *V*_2_. The relative phases (*φ*_2_ − 2 · *φ*_1_) cluster near −π/2 for CF motion (Figure 2C, orange), indicating a steep rise (inset in Figure 2C). CP motion typically evoked smaller-amplitude harmonics, i.e., a more sinusoidal waveform; the broad distribution of *φ*_2_ − 2 · *φ*_1_ (Figure 2C, blue) indicates a lack of correlation between fundamental and second-harmonic phases for CP motion. (Note that only cells with *V*_2_ ≥ 0.3 mV were included in the histogram, since phase measurements become meaningless for amplitudes close to or below the noise level.) There was a small but significant (*n* = 83, *p* ≤ 0.01; Figure 2D) difference in *V*_0_ between CF (0.7 ± 0.2 mV) and CP (1.1 ± 0.2 mV) stimuli. However, despite a smaller *V*_0_, CF motion evoked larger peak depolarizations than CP motion (Δ*V*_Peak_ [CF − CP] = 1.4 ± 0.2 mV; *n* = 26). Also note that voltage excursions might well be substantially larger in the dendrite than in the soma (see also Discussion).

To facilitate comparisons, we used directional asymmetry indices (*AI*_1_, *AI*_2_, and *AI*_3_; see Materials and Methods). In almost all cells, CF motion evoked larger fundamental responses (*AI*_1_ > 0 in 90% of the cells; Figure 2E) with higher harmonic content (*AI*_2 _> 0 in 80%, *AI*_3 _> 0 in 73% of cells; Figure 2E). The peak positions of the Gaussian fits to the *AI* distributions are (*n* = 83 cells): *AI*_1_ = 0.13 ± 0.01, *AI*_2_ = 0.25 ± 0.04, and *AI*_3_ = 0.22 ± 0.07. In order to check for the involvement of Cs^+^-sensitive K^+^ channels, we replaced intracellular K^+^ by Cs^+^ and found an increase in resting potential (with K^+^: *n* = 58, *V*_Rest_ = −62 ± 1 mV; with Cs^+^: *n* = 25, *V*_Rest_ = −48 ± 1 mV) but little effect on DS (with K^+^: *n* = 58, *AI*_1_ = 0.13 ± 0.01, *AI*_2_ = 0.27 ± 0.03, and *AI*_3_ = 0.19 ± 0.05; with Cs^+^: *n* = 25, *AI*_1_ = 0.14 ± 0.02, *AI*_2_ = 0.17 ± 0.07, and *AI*_3_ = 0.28 ± 0.01).

### Stimulus Contrast

**Figure 3:**
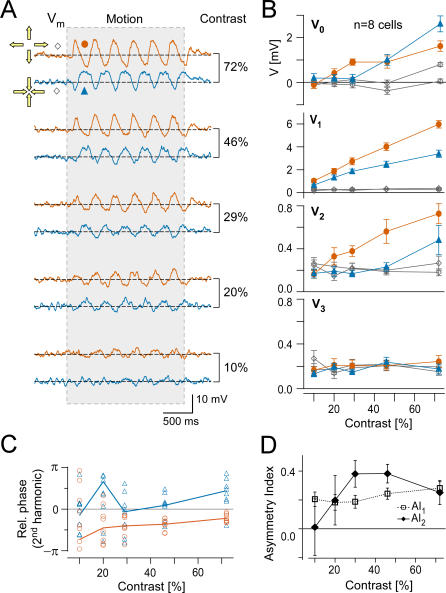
Contrast Dependence (A) Voltage responses evoked by circular wave stimuli (3 Hz; period: 192 μm, contrasts from 10% to 72%); traces are averages of three trials; CF motion in orange, CP in blue (*V*_Rest_ = −63 mV). Gray area indicates motion duration. (B) Amplitudes of DC (*V*_0_)*,* fundamental (*V*_1_)*,* and harmonics *V*_2_ and *V*_3_ as a function of stimulus contrast (average of eight cells). Prestimulus data (no-motion; open gray symbols) included. (C) Relative phases of second harmonic (*φ*_2_ − 2 · *φ*_1_) as function of contrast (same cells as in [B]). (D) *AI*s for *V_1_* (open squares) and *V_2_* (filled diamonds) from (B). For statistics, see Table 1

The response of a nonlinear system can depend on the stimulus amplitude in a much more complicated way than the simple scaling that is characteristic of a linear system. We therefore varied the stimulus contrast and found that *V*_1_ grew almost linearly with contrast (Figure 3A and 3B), albeit with different slopes for CP and CF motion. *V*_2_ for CP motion was indistinguishable from the (no-motion) background for all but the largest contrast (72%). For CF motion, *V*_2_ increased roughly linearly with increasing contrast and *φ*_2_ − 2 · *φ*_1_ became more tightly clustered near -π/2 (Figure 3C). *V*_0_ increased with contrast but with no consistent DS.

To rule out that the observed response asymmetry is restricted to a small velocity range we measured the dependence of the harmonic amplitudes on stimulus velocity (Figure S1). With a 4-fold change in speed (from 0.5 to 2 mm/s), *V*_0_, *V*_1_, and *V*_2_ varied only little; *V*_3_, however, fell by a factor of almost three, presumably due to capacitive filtering at higher frequencies.

### Inhibitory Inputs

SACs receive direct and indirect inhibitory synaptic input [35] from other cells in the circuitry. The direct inputs include inhibitory interactions between SACs [36], which involve GABA acting on GABA_A_ receptors [37], and glycinergic input [38] from other types of amacrine cells. The indirect inhibition results from GABA acting on GABA_C_ receptors at bipolar cell axon terminals [39,40], which provide SACs with glutamatergic excitatory input. Lateral inhibitory inputs are involved in the generation of DS SAC responses when image motion includes the surround [24].

**Figure 4:**
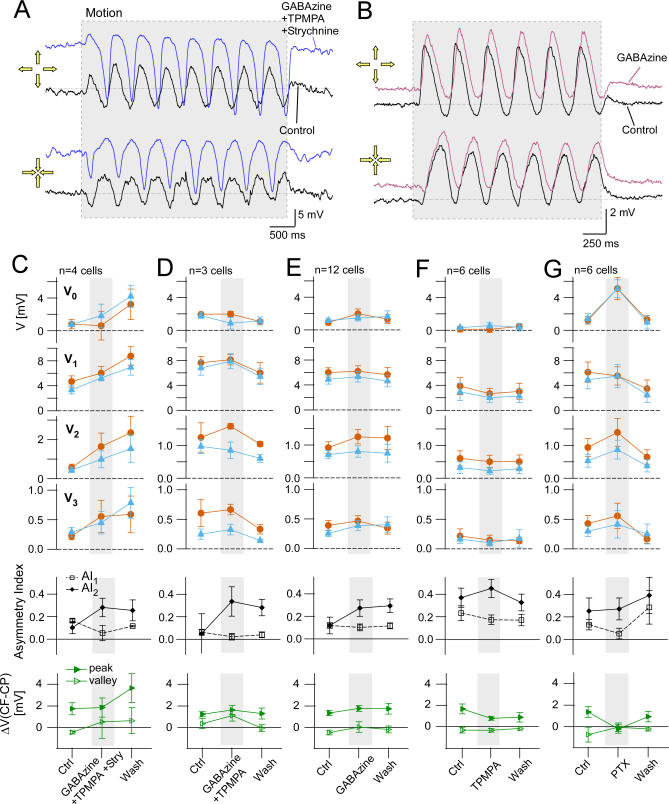
Effects of GABA Receptor Antagonists (A and B) Voltage responses to circular wave stimuli recorded before (control, black; *V*_Rest_ [A] −62 mV, [B] −49 mV) and during bath application of a mixture of GABA_A_, GABA_C_, and glycine receptor antagonists ([A] blue traces; Gbz+TPMPA+strychnine; *V*_Rest_ = −53 mV) and Gbz alone ([B] purple traces; *V*_Rest_ = −48 mV). Traces (averages of three trials) overlaid to facilitate comparison; the vertical shifts reflect drug-induced changes in *V*_Rest_ *;* (A) and (B) show different cells. Gray area indicates motion duration. (C–G) Top row: averaged amplitudes of DC (*V*_0_)*,* the fundamental (*V*_1_), and the harmonics (*V*_2_ and *V*_3_) for different GABA/glycine receptor antagonists and mixtures thereof (25–50 μM Gbz, 50–75 μM TPMPA, 300 μM PTX, 1 μM strychnine; CF motion: orange circles; CP motion: blue triangles; gray box: during drug application; stimuli: 2–3 Hz, 57%–72% contrast, stimulus period 192 or 256 μm). Middle row: *AI*s for fundamental (open squares) and second harmonic (filled diamonds). Bottom row: potential differences between CF and CP motion responses for depolarizing peaks and hyperpolarizing valleys. For statistics, see text and Table 1. Ctrl, control.

To test whether inhibitory input is required for the generation of DS SAC responses evoked by circular wave stimuli that are spatially confined to the SAC's dendritic arbor, we tested the effect of a mixture of selective antagonists that block GABA_A_, GABA_C_, and glycine receptors (i.e., GABAzine [Gbz], TPMPA, and strychnine, respectively). Even with a full cocktail of inhibitors, which eliminates all lateral interactions [24], the electrical response remained strongly DS (Figure 4A) for *V*_2_ and peak responses (Δ*V*_Peak_); only *V*_1_ DS was reduced (Figure 4C; Table 1). The depolarization of the resting potential (*V*_Rest_) that is also seen is consistent with blocking tonic inhibitory input. Similar results were obtained when applying Gbz and TPMPA together (Figure 4D). No significant reduction in the DS of *V*_1_, *V*_2_, or Δ*V*_Peak_ was found with either Gbz (Figure 4B and 4E), which selectively disrupts the SAC-to-SAC GABAergic inhibitory network, or TPMPA (Figure 4F), which selectively abolishes GABA-mediated presynaptic inhibition. *AI*_2_ increased, but not significantly, under both Gbz and TPMPA.

We also applied the widely used but nonspecific GABA-receptor antagonist picrotoxin (PTX) at a concentration (≈300 μM) reported to abolish GABA-induced currents in SACs (see Figure 4D and 4E in [38]). At this concentration, PTX also affects glycine receptors [41] and therefore should have an effect similar to a mixture of Gbz, TPMPA, and strychnine. PTX, like the Gbz+TPMPA+strychnine mixture, had no effect on *V*_2_ DS, but unlike the mixture, decreased Δ*V*_Peak_ (Figure 4G). PTX, but not the mixture, also caused an increase in *V*_0_ for both CF and CP stimuli—the only blocker to have a consistent effect on *V*_0_. The reason for this difference in the actions of these antagonists is not clear. That the effects of PTX, a nonselective antagonist, are different than the effects of a combination of selective blockers, suggests that PTX's actions are not restricted to GABA_A_, GABA_C_, and glycine receptors.

In the presence of blocker mixtures (Gbz+TPMPA or Gbz+TPMPA+strychnine), dendritic [Ca^2+^] is very unstable, with frequent large and spontaneous transients (X. Castell, unpublished data). This makes it impossible to reliably monitor the DS Ca^2+^ signaling when using our circular wave stimuli that have a physiologically realistic contrast and intensity.

In summary, the results with inhibitory blockers clearly demonstrate that a moving (circular wave) stimulus that is confined to the spatial extent of the cell can produce a DS electrical response that is independent of lateral inhibitory interactions. This shows that under these conditions, DS signaling in SACs is generated by a dendrite-intrinsic mechanism, which is investigated further in the following experiments.

### Nonlinearities Vary with Membrane Potential

**Figure 5:**
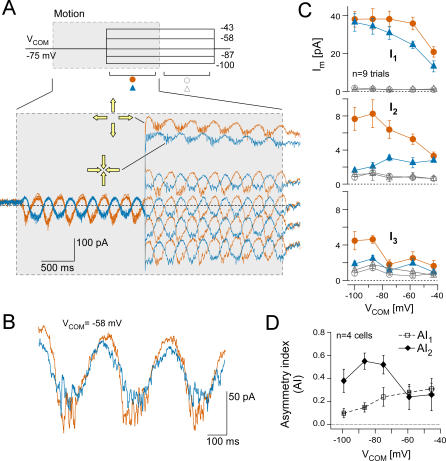
Voltage Dependence (A) Whole-cell voltage-clamp current responses evoked by circular wave stimuli (3 Hz; 46% contrast; period: 192 μm; averages of three trials; Cs^+^-based intracellular solution) while the somatic potential (*V_COM_*) was stepped from −75 mV to the potentials indicated. Stimulation sequence was repeated for different step potentials after waiting for 2 s. Gray area indicates motion duration. (B) Traces for CF motion (orange) and CP motion (blue) from (A) (for the voltage step to −58 mV) magnified and aligned (shifted by 167 ms) to illustrate difference in waveforms. (C) Amplitudes of the fundamental (*I*_1_)*,* and the second and third harmonics (*I*_2_, and *I*_3_) as a function of *V*_COM_ (analysis as in Figure 2.; averages of nine trials; same cell as in [A] and [B]). Motion (orange/blue-filled symbols) and no-motion (gray open symbols) data, both during voltage steps, are plotted. (D) *AI*s for *V*_1_ (open squares) and *V*_2_ (filled diamonds) as functions of *V*_COM_.

Voltage-gated channels (VGCs) have a limited and channel type–specific activation region. Thus, varying the somatic voltage (*V*_COM_) can help to test whether VGCs are involved in DS and/or response nonlinearity, and may help in the identification of the ion channel(s) involved. We measured light-induced current responses, which in shape and asymmetry resemble the voltage responses measured in current-clamp (Figure 5A and 5B), while stepping the somatic potential from −75 mV to voltages between −105 mV (below which cells became leaky) and −35 mV (Figures 5 and S2). Both fundamental (*I*_1_) and harmonics (*I*_2_ and *I*_3_) were substantially voltage dependent (Figure 5C). *I*_0_ is dominated by currents due to the voltage-step protocol and was not analyzed for DS. The direction sensitivities of *I*_1_ and *I*_2_ behave very differently as a function of step voltage (Figure 5C and 5D). At voltages negative to the resting potential (see Materials and Methods), the DS of *I*_1_ decreases, while the DS of *I*_2_ increases. The third harmonic (*I*_3_) is close to background (no-motion) levels around the resting potential but becomes substantially DS for strong hyperpolarization (Figure 5C).

Of all voltage dependencies analyzed, only that of *I*_1_ for CP motion (blue traces in Figures 5C and S2) resembles what would be expected from a passive dendrite, i.e., a linear *I–V* curve reversing at approximately 0 mV. Most other results (for CF motion *I*_1_, *I*_2_, and *I*_3_; and for CP motion *I*_2_) indicate the presence of a nonlinearity that depends on holding voltage, in a manner consistent with the presence of VGCs.

### Calcium Channels and Response Nonlinearity

The strong correspondence between motion-induced [Ca^2+^] transients and nonlinearity in the voltage responses (Figure 1E) suggests a role for VGCCs, which are present in SACs [42]. Furthermore, various Ca^2+^ channel blockers abolish DS in DSGCs while leaving light responsiveness largely intact [43]. We confirmed this effect of Cd^2+^ with recordings from DSGCs (unpublished data), albeit at concentrations of 20 μM rather than the 60–110 μM used by Jensen [43].

**Figure 6:**
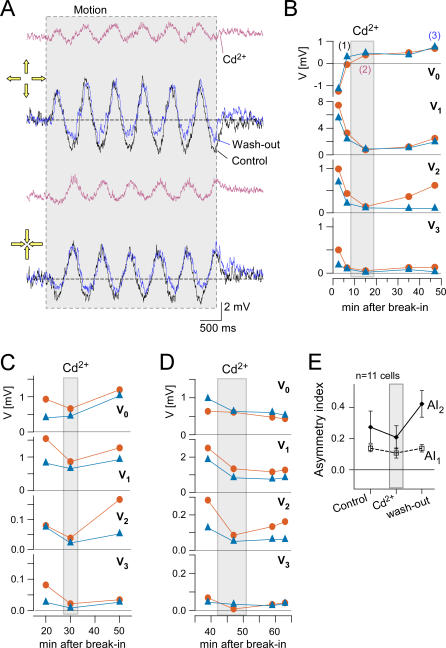
Effects of Cd^2+^ (A) Voltage responses to circular wave stimuli (3 Hz; 57% contrast; period: 192 μm) recorded before (control, *V*_Rest_ = −63 mV; black), during (*V*_Rest_ = −52 mV; purple) and after (washout, *V*_Rest_ = −63 mV; blue) bath application of 10 μM Cd^2+^. Traces (averages of three trials) are overlaid to facilitate comparison. Gray area indicates motion duration. (B–D) Amplitudes of DC (*V*_0_), fundamental (*V*_1_), and harmonics (*V*_2_ and *V*_3_) plotted over time (in minutes after break-in; CF motion: orange circles; CP motion: blue triangles). Three different cells are shown ([B] shows the data from the cell in [A]; traces in [A] are at 6.5, 15, and 47 min after break-in). (E) *AI*s for *V*_1_ (open squares) and *V*_2_ (filled diamonds). For statistics, see Table 1.

Because the loss of DS in ganglion cells could result either from the suppression of synaptic inputs from SACs or from the block of DS generation in SAC dendrites, we measured the effect of Cd^2+^ on SAC responses. Both *AI*_1_ and *AI*_2_ were reduced by 10 μM Cd^2+^ (Figure 6; Table 1). At a higher concentration (90 μM), Cd^2+^ eliminated SAC light responses altogether (six cells, unpublished data), presumably by blocking synaptic pathways. The effects of Cd^2+^ were variable (Figure 6B–6D), possibly because the concentration range that leaves the synaptic input from bipolar cells intact is narrow. In experiments with other VGCC blockers, such as ω-conotoxin M7C (reviewed in [44]), the inputs to SACs were either strongly reduced or the DS was unaffected (unpublished data).

### Dendritic [Ca^2+^] Changes in Response to Voltage Steps

**Figure 7:**
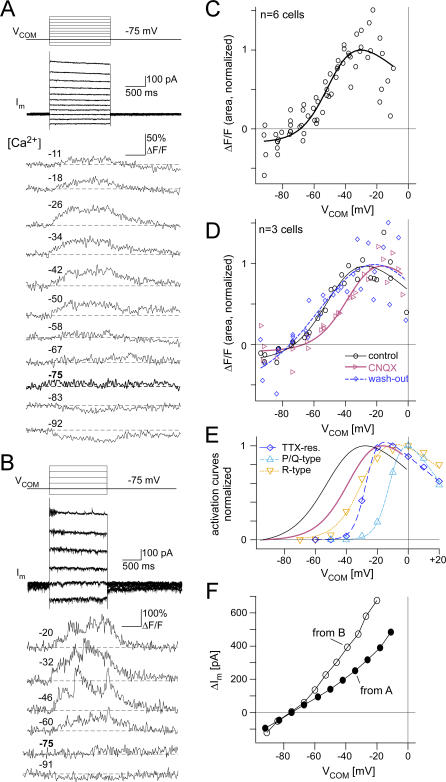
Voltage-Dependent Dendritic Ca^2+^ Signals (A) Somatic voltage step (from *V*_COM_ = −75 mV) evoked changes in somatic current (*I*_m_) and dendritic [Ca^2+^] (single trials). Oregon-Green 488 BAPTA-1 (100 μM) signals (Δ*F/F* in percentages) recorded by two-photon imaging; Cs^+^-based intracellular solution. (B) Example of spiky [Ca^2+^] transients riding on smooth responses (different cell but same protocol as in [A], single trials; some traces omitted). (C) Ca^2+^ responses (circles) as a function of *V*_COM_ for cells lacking spiky activity; each symbol represents a single response; for each cell, amplitudes were normalized to the response-amplitude at −35 mV (see Materials and Methods); continuous line: fitted activation curve (using Equation 5). (D) [Ca^2+^] versus voltage data before (control; black) during (purple) and after (washout; blue) bath application of CNQX (10 μM). (E) Curve fits from C and D overlaid with fits (Equation 5) to data from the literature for P/Q-type (cyan upward triangles, *g* = −0.023 ± 0.001; *V*_50_ = −10.9 ± 0.5 mV; *V*_Slope_ = 4.56 ± 0.35; *E*_Ca_ = 46.8 ± 1.2 mV; [45] and R-type (orange downward triangles, *g* = −0.013 ± 0.003; *V*_50_ = −27.5 ± 2.1 mV; *V*_Slope_ = 8.57 ± 1.09; *E*_Ca_ = 81.2 ± 13.1 mV; [60] calcium channels, as well as for TTX-resistant Na^+^ channels (blue diamonds, *g* = −0.013 ± 0.0003; *V*_50_ = −26.9 ± 0.2 mV; *V*_Slope_ = 4.00 ± 0.15; *E*_Na_ = 66.2 ± 1.1 mV; [78]. (F) Somatic current as function of the step-to potential (filled circles: cell in [A], *I*(−75 mV) = −64 pA; open circles: cell in [B], *I*(−75 mV) = −86 pA).

**Figure 9:**
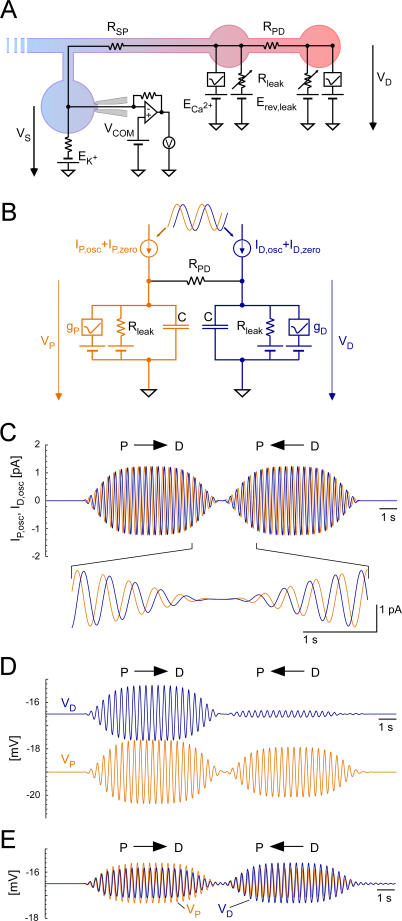
Two-Compartment Model of a Dendritic Branch of a Starburst Cell (A) Schematic of SAC electrotonics, illustrating the proposed gradient between dendritic and somatic voltages (*V*_D_ and *V*_S_; red represents relative depolarization; see also Equation 1); circuit components: longitudinal resistors between soma and proximal compartment (*R*_SP_) and between proximal and distal compartment (*R*_PD_)*,* distal leak resistance (*R*_leak_)*,* a voltage-gated Ca^2+^ and the somatic K^+^ conductance (with respective reversal potentials *E*_rev,leak_, *E*_Ca^2+^_, and *E*_K^+^_), and the patch-clamp amplifier. (B) The model consists of two identical dendritic compartments, P (proximal) and D (distal), that each contain in parallel a capacitor (C), a leak resistance (*R*_leak_) and a Hodgkin-Huxley–type voltage-gated conductance with identical properties in both compartments (*g*_P_ or *g*_D_). The compartments are connected by a resistor (*R*_PD_). The inputs from bipolar cells are represented by sinusoidal currents (*I*_P,osc_ and *I*_D,osc_) that are injected into the compartments. The resting potentials are set by additionally injecting DC currents (*I*_P,zero_ and *I*_D,zero_). Image motion is simulated by setting the relative phase of the sinusoidal currents injected into the two compartments to either +90 or −90 degrees. For equations, see Materials and Methods. We analyzed the direction discrimination of the model using *R*_PD_ = 1 GΩ, *C* = 183 pF, *g*_open_·*n*_ch_ = 5 nS, *R*_leak_ = 160 MΩ, and a VGC with *E*_rev_ = 50 mV, *k*_m_ = 1,000/s, *k*_h_ = 7.25/s, *V*_m50_ = −15 mV, *V*_h50_ = −15 mV, *V*_mSlope_ = 4.5 mV, and *V*_hSlope_ = −4 mV. Although the channel parameters do not apply to a specific type of VGC, they are in the range that is plausible for the types of Ca^2+^ channels found in SACs. To mimic a dendritic voltage gradient, the resting potential of the two compartments were set to −19 and −16.5 mV, by setting *I*_P,zero_ = 195 pA and *I*_D,zero_ = 183 pA (outward currents). This configuration is stable, i.e., in the absence of sinusoidal input currents the voltage remains steady and behaves largely linearly, as shown by numerical simulation. (C) Sinusoidal input currents (with a frequency of 2.86 Hz and an amplitude of 1.25 pA) injected into the two compartments. During the first stimulation period, the current injected into compartment D is delayed relative to compartment P (simulating “CF” motion, from P to D). During the second stimulation period, the timing is reversed (simulating “CP” motion, from D to P). (D) Voltages in both compartments in response to the injection of oscillating currents from (C). Amplitudes are given as root mean square at the fundamental frequency. In both compartments, the response is larger when the current in the compartment that has the more depolarized resting potential (−16.5 mV), in this case compartment D, is delayed. It is this compartment that shows a strong (1.09 mV vs. 0.13 mV, i.e., ≈8.5-fold larger) preference for one “motion direction” (blue trace). The other compartment has a much weaker preference (1.26 vs. 0.98 mV, i.e., ≈1.3-fold) but for the *same* direction (orange trace). For details, see Table 2. (E) If the injected DC current was set such that both compartments rest at the same potential, e.g., at −16.5 mV, the two compartments prefer different directions.

To test whether distal dendritic VGCCs are active near the resting potential, we measured changes in dendritic [Ca^2+^] during somatic voltage steps (Figure 7). The Ca^2+^ signals reflect local VGCC activity, whereas when measuring whole-cell currents (as in [42]), the locations of the activated channels are unclear, and currents due to channels electrotonically distant from the recording can be severely filtered. Substantial voltage-evoked Ca^2+^ responses (Figure 7A) were observed in a majority of cells (20 out of 27). Some of these cells (seven out of 20) displayed large, spiky [Ca^2+^] transients riding on top of smooth responses (Figure 7B). Light-induced Ca^2+^ responses (for which 12 of the 20 cells were tested) were found more often (three of five) in SACs with spiky transients than in cells without them (one of seven). We constructed an activation curve (Figure 7C) using only SACs without spiky transients (compare Figure 7A and 7B) and fitted it with a channel activation curve (Equation 5, Materials and Methods), finding a half-activation voltage *V*_50_ = −49 ± 2 mV and a slope voltage *V*_Slope_ = 8.7 ± 2.0 mV (*n* = 6 cells; other fit parameters: *A*_Ca,0_ = −0.17 ± 0.08; *g* = −0.016 ± 0.002). *V*_50_ is much lower and *V*_Slope_ is much larger than the nominal values for high-voltage–activated (HVA) VGCCs found in SACs [42] (e.g., for P/Q-type: *V*_50_ ≈ −11 mV, *V*_Slope_ ≈ 4.56 mV, from data in [45]). This could, for example, be due to different, i.e., low-voltage–activated (LVA), types of VGCC in the distal dendrites. That T-type or LVA L-type channels [46] contribute significantly is unlikely because SAC Ca^2+^ currents are not sensitive to Ni^2+^ [42], which blocks T-type channels more effectively than other Ca^2+^ channels [47], or nifedipine, which inhibits L-type channels [42]. Another possibility is a dendritic voltage gradient, which is, in fact, plausible since SAC dendritic branches are rather thin [21]. The requisite steady radial current, outward at or near the soma and inward near the tips (Figure 9A), could flow out through K^+^ channels that are open at the resting potential (and are not completely blocked by intracellular Cs^+^ [48]) and in through channels with a more positive reversal potential, such as glutamate-gated channels, which are tonically activated on SACs [35]. Since glutamate receptors are preferentially located on branch points and varicosities [49], glutamatergic input should, in fact, be stronger distally, where it is also more effective because the somatic current-sink [20] shunts proximal inputs.

**Table 2:**
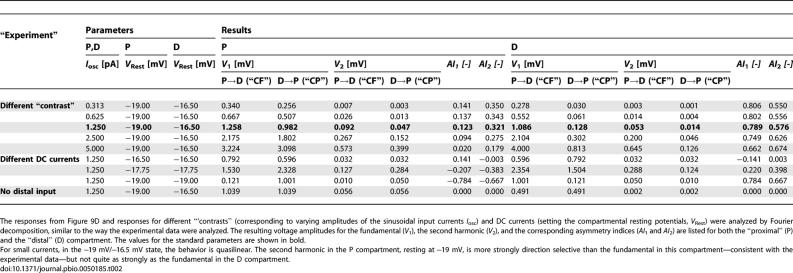
Performance of the Two-Compartment Model

An antagonist of AMPA-type glutamate receptors, CNQX, shifted the [Ca^2+^] versus *V* curve towards depolarized potentials by 15 mV (Figure 7D; *n* = 3 cells; fit parameters: *V*_50_ = −34 ± 3 mV; *A*_Ca/0_ = −0.09 ± 0.06; *g* = −0.019 ± 0.003; *V*_Slope_ = 10.2 ± 2.2 mV, see Equation 5) and reduced the holding current (from −96 ± 2 pA to −76 ± 9 pA at *V*_COM_ = −75 mV, *n* = 5; and from −186 ± 7 pA to −141 ± 5 pA at *V*_COM_ = −83 mV; *n* = 3).

This reduction, which confirms the tonic activation of the AMPA-type receptors, may be considerably smaller than the actual tonic current through AMPA-type channels, which is partially compensated due to reduced GABA-receptor–mediated current into SACs because CNQX blocks excitatory input onto inhibitory interneurons (other amacrine cells). The current carried by NMDA-type glutamate receptors in SACs [38] is likely too small to play a major role.

### Radial Location of the Motion Detector

**Figure 8:**
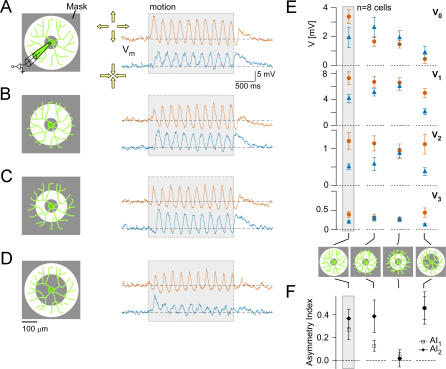
Stimulus-Coverage Dependence (A–D) Voltage responses to circular wave stimuli (3 Hz; 72% contrast; period: 192 μm) that cover different areas of the SAC dendritic field (see Materials and Methods; averages of three trials; CF motion in orange, CP in blue). Left column: stimulus masks, white areas indicate where the moving stimulus is presented (*V*_Rest_ = −62 mV). (E) Amplitudes of DC (*V*_0_)*,* fundamental (*V*_1_)*,* and harmonics (*V*_2_ and *V*_3_) plotted versus mask configuration (averages of eight cells). Leftmost data (gray box): “standard” stimulus from (A). (F) *AI*s for *V*_1_ (open squares) and *V*_2_ (filled diamonds) from (E). For statistics, see Table 1.

Finally, we examined where along the dendritic branches the moving stimulus has to be presented in order to elicit DS nonlinearities. To do this, different regions of circular wave stimuli were masked by concentric gray rings (Figure 8). Reducing the stimulus diameter (Figure 8B, 8C, and 8F) led to a reduction in DS, mostly due to an increase in the amplitude for CP motion. Responses to CP and CF stimuli became more similar (Figure 8E and 8F) for both fundamental (*V*_1_) and harmonic responses (*V*_2_ and *V*_3_). When the masked region in the center was enlarged (Figure 8D), DS increased for *V*_1_, *V*_2_, and *V*_3._ The DC component (*V*_0_) dropped to nearly zero when most of the center region was masked (Figure 8D and 8E), with no clear DS in any except the “standard” configuration (Figure 8A, 8E, and 8F).

Taken together, this suggests that the motion-response asymmetry is computed in the distal section of the dendrite, which is consistent with the radial location of the intensity gradient at the time when voltage and [Ca^2+^] rise steeply (Figure 1E). The decrease in *V*_1_ DS for smaller stimulus diameters is expected as a result of a reduction of distal input (see model), but a reduction in lateral inhibition may be a contributing factor. The finding that *V*_2_ DS, which does not depend on inhibition, also decreases with stimulus diameter highlights the importance of the distal region for dendritic DS computation.

## Discussion

Although a number of studies (reviewed in [26,50]) have shown that SACs are necessary for the computation of motion direction in the retina and that SAC dendrites generate direction-dependent signals [15,24], it is still not well understood how the DS signal is actually computed. In this study, we have focused on dendritic electrical mechanisms and found experimental and modeling evidence that they are, by themselves, able to generate DS signals, in the absence of lateral inhibitory interactions, and are thus almost certain to play an important role in generating DS synaptic output from SACs.

### Inhibitory Network Interactions

Based mainly on two observations, it has been proposed [14] that the computation of SAC DS requires lateral synaptic inhibition. First, blocking GABAergic inhibition abolishes DS responses (but not general responsiveness to light) in DSGCs [51–53], which may, however, simply result from blocking DS input into DSGCs. Second, anatomical ([21,54] but see [10]) and physiological [24,36] evidence indicate GABAergic inhibition between SACs. Recent modeling even suggests that reciprocal inhibitory interactions in the SAC network could be sufficient to create SAC DS [55].

Surround stimulation inhibits SACs [30,35] and depresses dendritic [Ca^2+^] levels [15,24]. Thus, it was surprising that neither surround inhibition nor DS of dendritic Ca^2+^ signals were abolished by blocking GABA_A_ receptor–mediated transmission [15]. To completely block surround effects on the dendritic Ca^2+^ signals, simultaneously blocking GABA_A_, GABA_C_, and glycine receptors is required [24]. This indicates that GABA- and glycine-mediated lateral inhibition can contribute to dendritic DS in SACs.

Our pharmacological data show, however, that for stimuli with movement constrained to the SAC dendritic field, DS persists almost unchanged when the direct, GABA_A_ receptor–mediated [24] connections between SACs are blocked. Only with all lateral inhibitory interactions blocked, which also leads to substantial tonic depolarization, is DS affected (Figure 4E and 4F). Consistent with Lee and Zhou [24] the response to CP motion (*V*_1,CP_) increases (Figure 4E–4G), leading to a reduced *V*_1_ DS. It is, furthermore, inconsistent with a lateral-inhibition–based mechanism that *V*_0,CP_ (the DC response for CP motion) is larger than *V*_0,CF_ (Figure 2D), because stronger inhibition for CP motion should to lead to a smaller V_0,CP_. This and our pharmacological results suggest that an inhibition-independent mechanism is used for DS detection of movement inside the dendritic field (reminiscent of the CF facilitation observed by Lee and Zhou [24]). There may be additional (inhibition-based) mechanisms used for stimuli that extend into the surround [24]. That *V*_1_ DS for dendritic field–restricted stimuli is reduced by blocking GABA_A_, GABA_C_, and glycine receptors may be caused by an indirect effect of the strong (as much as 8 mV) shift in resting potential, rather than the interruption of lateral inhibition. An absolute requirement of lateral inhibitory network interactions for DS generation is ruled out by the undiminished presence of *V*_2_ DS and Δ*V*_Peak_ DS under these conditions.

### Direction Selectivity at the Fundamental Frequency

That the somatically measured fundamental-frequency voltage and current components (*V*_1_ and *I*_1_) are larger for CF than for CP stimuli (Figure 1D and 1E, and [15]) contradicts model predictions [23,29]. One possible explanation is that SACs receive DS synaptic input. If excitatory inputs were DS, the difference in the amplitude of the fundamental response for CP and CF stimuli should increase with hyperpolarization, i.e., increased driving force (light-evoked synaptic currents are nonrectifying in SACs [35]). Instead, *I*_1_ DS becomes smaller at more-negative potentials (Figures 5C and 5D). For inhibitory inputs, the situation is more complicated. Although the voltage dependence of *I_1_* DS is consistent with that of direct inhibitory synaptic inputs, such as those resulting from SAC–SAC interactions [36], *V*_1_ DS persists in the presence of GABA_A_ blockers. Indirect inhibition via presynaptic GABA_C_ receptors has the voltage dependence of glutamatergic currents. The reduction of *V*_1_ DS by blocking GABA_A_, GABA_C_, and glycine receptors might alternatively be due to changes in the center-surround structure of bipolar cells [56] or due to changes in the active electrical mechanism (discussed next) as a result of the depolarization caused by the elimination of tonic inhibition.

An alternative explanation for the DS at the fundamental frequency is a dendrite-intrinsic direction-dependent amplification of synaptic input currents by activation of VGCs. One way to picture such signal amplification is that the negative slope conductance [57] that occurs in a certain part of the VGCs' activation range compensates some of the native (including synaptic) conductance and thus increases the input resistance. This in turn leads to a larger (amplified) voltage change for a given input current. For significant amplification, the negative slope conductance needs to be comparable to the passive conductance. SACs possess N-, P/Q-, and R-type Ca^2+^ channels [42], with N- and P/Q-type channels contributing approximately 70% of the Ca^2+^ current. In addition, SACs may express TTX-resistant voltage-gated Na^+^ channels (B. J. O'Brien, personal communication). We used P/Q-type channel data from the literature [45,58] and estimated (see Materials and Methods) the size of the slope conductance provided by these VGCCs. For approximately 930 channels, which are needed to account for *I*_2_ (see below), the negative slope conductance amounts to approximately 1/(−130 MΩ). This value is comparable to the light-induced synaptic conductance (≈3.7 *nS* = 1/(270 MΩ) deduced from Figure 4B in [35]). The slope conductance depends strongly on the voltage (location on the activation curve; Figure 7E) and becomes negligible below the activation range. This means that no VGC-based amplification should occur at very hyperpolarized potentials, which is consistent with the observed dependence of fundamental frequency DS on the holding voltage (Figure 5C and 5D).

### Electrical Nonlinearity and Voltage-Gated Channels

The presence of a second harmonic component (*V*_2_) even for lower-contrast CF motion (Figure 3) indicates that an electrical nonlinearity is involved in SAC DS. The clustering of the relative phases (*φ*_2_ − 2 · *φ*_1_) between fundamental and second harmonic at negative values around −π/2 (Figure 2C) shows that, even when it is not obvious from the response trace, nonlinearity occurs mainly during the rising phase of the fundamental. This and the steep rise in the voltage and [Ca^2+^] for CF motion (Figure 1E) suggest that the distal-dendritic resting potential might hover near the threshold of regenerative events (e.g., Ca^2+^ spikes). This might also be the reason that action potentials have been seen in some [59], but not all ([15,30,35] and present study), SAC electrical recordings. Presynaptic pathways do not appear to contribute substantially to the response nonlinearity, since only at the highest contrasts do the values of *V*_2_ for CP motion rise substantially above background (Figure 3B).

As mentioned above, HVA VGCCs are likely responsible for the nonlinearity. Therefore, it was somewhat surprising that the measured *I*_2_ shows only a shallow maximum (Figure 5C) as a function of the somatic holding potential, since we initially expected that hyperpolarization beyond the activation range of these channels would suppress the nonlinear current component completely. That this was not possible, within the range of holding potentials that the cells would tolerate, indicates that there is voltage attenuation between soma and dendrite (see next section). We can, however, estimate whether the number of Ca^2+^ channels needed to generate the second harmonic signals we see is consistent with Ca^2+^ currents reported by Cohen [42] by assuming moderate voltage modulation amplitudes and expanding the *I–V* curve into a Taylor series (see Protocol S1). If we neglect inactivation and kinetic aspects, and assume a modulation amplitude (*V*_A_) of 5 mV (Figure 1D), we find that approximately 930 channels are needed to get *I*_2_ = 8 pA (Figure 5C). To generate the Ca^2+^ conductance seen in SACs (≈4 *nS*, [42]), we need approximately 300 channels. (This takes into account that Cohen used 110 mM Ba^2+^, which increases the single-channel conductance by a factor of approximately three, see [58].) The discrepancy in channel-number estimates might simply reflect the fact that they are based on measurements from different preparations and are affected differently by dendritic voltage attenuation and current shunting. If, for example, the voltage modulation in the dendrite (*V*_A_) is twice that measured in the soma and even if the current from the dendrite (*I*_2_) is attenuated by the same factor, which is greater shunting than is likely, the number of channels (Protocol S1, Equation S6) would be half as large (465) and thus in closer agreement with the number estimated using Cohen's data [42]. That voltage excursions in the distal dendrite (where, owing to the branching pattern, one finds most synaptic inputs) are larger than in the soma is likely, because graded potentials decrement as they spread electrotonically towards the soma.

### Membrane Potential and [Ca^2+^] in the Distal Dendrites

One problem when investigating dendritic processing is that it is not only impossible to record electrically from most of the dendrite, but it is also difficult to control the voltage in parts of the dendrite that are remote from the electrode location (the “space clamp” problem). This is particularly acute in SACs, in which the dendritic connections between the soma and distal branches are very thin [8], and synapses are tonically activated (Figure 7D and [35]). We therefore need to consider explicitly the voltage attenuation and shift due to the dendritic voltage divider (Figure 9A).

Earlier Ca^2+^ measurements from SAC dendrites [15] showed that at rest (i.e., with constant, uniform illumination), there is a steady-state Ca^2+^ influx that can be transiently reduced by suitable stimuli. The [Ca^2+^] versus voltage-step data (Figure 7) confirm this since they show that the dendritic voltage is still within the activation range of VGCCs when clamping the soma at the zero-current potential (*V*_Rest_). Because LVA Ca^2+^ channels have not been found in SACs [42], it is conceivable that the Ca^2+^ influx is through HVA channels and the measured Ca^2+^ activation curve (Figures 7E) has been shifted by attenuation of the somatic control voltage via a longitudinal dendritic resistance (*R*_PD_) [16] in combination with a distal leak conductance (1/*R*_leak_) that has a positive reversal voltage (Figure 5E). Note that the shape of the activation curve for SAC–SAC synaptic currents (Figure 1E in [24]) is consistent with the presence of such a dendritic voltage divider.

We can roughly estimate the properties of the dendritic voltage divider (Figure 9A) by comparing the activation parameters (*V*_50_ = −48.5 mV, *V*_Slope_ = 8.7 mV) obtained from the [Ca^2+^] versus *V* data (Figure 7) to those measured under proper voltage clamp. For P/Q-type channels [45], we estimate *V*_50_ = −10.9 mV and *V*_Slope_ = 4.56 mV, and for R-type channels [60], *V*_50_ = −27.5 mV and *V*_Slope_ = 8.57 mV. (Because their activation parameters are sufficiently similar, we lumped N- and P/Q-type channels together.) A combination of 70% P/Q-type and 30% R-type channels (see above) is fit approximately using *V*_50_ = −10.4 mV and *V*_Slope_ = 8.3 mV. The relation between distal voltage (*V*_D_) and the somatic voltage (*V*_S_) is

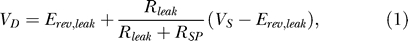
where *E*_rev,leak_ is the reversal potential of the distal leak. Changes in the somatic voltage reach the dendritic tips attenuated by the factor γ = *R*_leak_/(*R*_leak_ + *R*_SP_); it can be shown that, therefore, the apparent *V*_Slope_ is increased by 1/γ. For the P/Q-type channel, γ = 4.56/8.7 = 0.524, while for the R-type channel, γ would need to be 0.985, and for the combined current, γ = 8.3/8.7 = 0.95. To get *E*_rev,leak_, we can set *V*_S_ to the apparent half-activation voltage (*V*_50S_) and *V*_D_ to the actual half-activation voltages (*V*_50VC_) and use Equation 1 to find *E*_rev,leak_ = (*V*_50VC_ − γ*V*_50S_)/(1 − γ), which, obviously, becomes very error prone as γ approaches one. For the P/Q-type and the combined currents, we find *E*_rev,leak_ = 30.5 mV and *E*_rev,leak_ = 780 mV, respectively.

The combined currents lead to rather unlikely values for both γ and *E*_rev,leak_, which may mean that R-type channels are not involved in distal Ca^2+^ influx. For P/Q-type channels, the γ value is reasonable, but the leak reversal potential seems too high. We have not, however, taken into account: (1) persistent currents [61] through VGCCs or voltage-gated Na^+^ channels (B. J. O'Brien, personal communication), which steepen the activation curve and shift *E*_rev,leak_ to more positive potentials, because as these VGCs get activated, the ensuing current depolarizes the dendrite, leading in turn to an accelerated activation of VGCs. This could account for the otherwise implausible value of γ ≈ 1 for the R-type and the P/Q-R mixture; (2) The channel activation curves used for comparison (Figure 7E) are measured using Ba^2+^, which not only shifts the activation range of VGCCs to more positive values (e.g., [60]), but also increases the open-channel conductance (reviewed in [62]), potentially increasing the slope of the activation curve under conditions of imperfect voltage clamp. Please note that we have here lumped together the two dendritic compartments shown in Figure 9A, because, due to the larger number of distal branches, *R*_SP_ ≫ *R*_PD_.

In conclusion, the voltage-divider (voltage gradient) hypothesis reconciles the voltage-step–evoked Ca^2+^ signals with the pharmacological evidence [42] for the predominance of HVA Ca^2+^ channels in SACs.

### Model of Dendritic DS in SACs

Published models of dendritic DS in SACs can be roughly divided into two classes. One requires lateral inhibition (e.g., [14,31,55]), which, through the interaction with excitatory input, can generate nonlinearity and substantial DS [14,33]. Although lateral inhibition can contribute to dendritic DS [24], even blocking GABA_A_, GABA_C_, and glycine receptor-mediated inhibition leaves signaling in SACs clearly DS (Figure 4F). Models in the other class rely on the passive electrical properties of the dendrite and show that moderate DS can be generated by the SAC's morphology alone (e.g., [23]), but predict somatic voltage excursions that are larger for CP than for CF motion, which does not fit the experimental data (Figure 1C and 1D, and [15,30,31]). Passive models (e.g., [23]) also do not generate enough discrimination to explain how the DS system as a whole [27] is able to prefer a substantially lower contrast, preferred-direction stimuli over higher-contrast, null-direction stimuli.

Our data suggest that active (voltage-gated) currents are involved in DS computation, but the question remains as to how. In order to explore this issue, we constructed a two-compartment model (representing, e.g., distal and proximal dendrites; Figure 9B) in which each compartment contains, in addition to a linear leak conductance and a capacitance, a single type of VGC, to which we gave the properties of HVA Ca^2+^ channels. This we did because HVA Ca^2+^ channels are present in SACs [42]; other VGC types should work as well, but we have not explored this here. The compartments are connected by a resistor, representing the longitudinal intradendritic current pathway. The moving stimulus is represented by the injection of phase-shifted sinusoidal currents (Figure 9C).

We found that for a certain set of parameters, even if both compartments are otherwise identical, a small difference in the resting potentials (e.g., −19 mV and −16.5 mV) between the compartments allows a strongly DS response, such that in the more depolarized (distal) compartment, the “CF” stimulus causes an approximately 8.5-fold larger (fundamental-frequency) voltage amplitude than the “CP” stimulus (Figure 9D). Consistent with the somatic voltage recordings (e.g., Figure 1C–1E), the more negative (proximal) compartment also prefers the CF stimulus but only moderately so (by factors of ≈1.3 for *V*_1_ and ≈1.9 for *V*_2_). Thus, the model explains why the somatic voltage and the dendritic [Ca^2+^] both prefer CF motion (Figure 1E). The model is also consistent with our experimental data in that (1) DS is stronger (Figure 2E) for the second harmonic, (2) DS is voltage dependent (Figure 5C and 5D), (3) DS is largely independent of stimulus contrast (Figure 3D), and (4) in the absence of distal stimulation, *V*_1_ for “CP” increases (Figure 8C; Table 2). In the distal model compartment, the responses to preferred-direction stimuli with weak contrasts (corresponding to small *I*_osc_) are larger than responses to null-direction stimuli with as much as 4-fold larger contrasts (Figure 9D; Table 2). These are important features that cannot be reproduced by passive models, and strengthen the case for a central role of active conductances in dendrite-autonomous DS. In this regard, the SAC DS mechanism resembles frequency selection in the rod-photoreceptor network [63] and in auditory hair cells [64]. We would like to emphasize that ours is a proof-of-concept model. Using the current parameters, significant DS for both *V*_1_ and *V*_2_ is found over a frequency (velocity) range spanning only about a factor of three (unpublished data). More detailed versions of this model (with more radial compartments and multiple types of channels) will be needed to explain the full range of experimental observations.

A central property of our model is that a strong directional preference is imposed on an otherwise symmetrical arrangement by a mild voltage gradient (compare Figure 9D and 9E), as it appears to be present in SAC dendrites (Figure 7, see also Figure 1E in [24]). Only a small fraction of the total voltage drop between dendrite and soma (most of which may occur in the proximal dendrite [16]) would be needed. This is consistent with our experimental finding (Figure 8D) that strong DS results when presenting motion to only the outer part of the dendritic arbor.

The dendritic voltage gradient thus generates the necessary spatial asymmetry for computation of DS, by causing the VGCs in the two compartments to operate in slightly different regimes. Although in the model the proximal compartment is slightly DS, strong DS is limited to the distal compartment, which is where it is needed to drive DS Ca^2+^ signals [15] and, in turn, DS synaptic release.

### Calcium-Induced Calcium Release

Amplification of Ca^2+^ influx by calcium-induced calcium release (CICR) has been proposed as a mechanism potentially well matched to the timing requirements for the detection of slowly moving stimuli [65]. Although the biochemical machinery necessary for CICR has been found in cultured GABAergic amacrine cells [66,67], these cells were not classified further, and it is currently unknown whether CICR is present in SACs. The abolition of light stimulus–induced [Ca^2+^] transients during most whole-cell recordings is not due to a loss of VGCC function, because depolarization can still evoke an increase in [Ca^2+^] (Figure 7A). Also, in retinal ganglion cells recorded under similar conditions, dendritic Ca^2+^ responses persist [68], suggesting the loss of light-induced Ca^2+^ responses to be SAC specific. CICR is potentially sensitive to washout of cellular components during whole-cell recording and might be required in SACs to generate detectable Ca^2+^ responses to light stimuli. The latter is supported by our observation that cells exhibiting spontaneous spiky [Ca^2+^] transients during depolarization (Figure 7B), which could reflect CICR activity, are also more likely to show light-induced Ca^2+^ responses.

The persistence of DS electrical responses (linear and nonlinear) in the absence of any light stimulus–induced [Ca^2+^] transients suggests, however, that DS computation as such is independent of CICR. It remains to be explained why, if Ca^2+^ fluxes underlie the nonlinear currents (Figure 5), they cannot be detected as changes in Ca^2+^-dependent fluorescence. During a 100-ms period (the rising phase of the stimulus), about 32 amol (atto-mol = 10^−18^ mol) of Ca^2+^ enters the cell (estimated α = 7.4 pS [slope conductance, see Protocol S1, Equation S3], 500 VGCCs, and Δ*V* = 10 mV). This is small compared to the total amount of Ca^2+^ indicator; approximately 320 amol, estimated using a volume of the SAC's dendrite of 3.2 pL (based on [16,21]) and [OGB-1] ≈ 100 μM; the change in fluorescence intensity with this amount of indicator would be much less than the light-induced changes actually seen [15].

Since DS Ca^2+^-current densities are thus expected to be rather small in absolute terms (even though the inward/outward ratios might still be quite large), it is possible that CICR is needed to amplify [Ca^2+^] changes to levels that are sufficient for driving synaptic output from SACs, which is Ca^2+^ dependent [36,69]. It may, in fact, be impossible to allow entry of sufficient Ca^2+^ through channels to drive release without increasing Ca^2+^ channel density into a regime in which the dendrite becomes electrically unstable. CICR, due to its lack of electrogenicity, would circumvent this problem. Finally, CICR is expected to be highly nonlinear and thus could contribute to the rectification of the DS signal.

### Conclusion

Information about image motion is computed in SAC dendrites. The persistence of DS signals in SACs in the presence of GABA and glycine receptor antagonists shows that SAC dendrites by themselves generate DS signals, in the absence of lateral inhibitory interactions. There is strong evidence that VGCs, probably Ca^2+^ channels, and a gradient in membrane potential between dendritic tips and soma are involved. As we show for a two-compartment model, VGCs, when combined with the voltage gradient, can provide electrogenic timing-, frequency-, and DS amplification of synaptic input currents. VGCCs could, in addition, produce either directly or via CICR the increase in local [Ca^2+^] necessary for synaptic release and thus for the transmission of DS signals to DSGCs. Dendrite-autonomous direction detection may thus be one of the most convincing examples yet for the power of dendritic computation (reviewed in [70]).

## Materials and Methods

### Animals and tissue preparation.

Experiments were done on adult New Zealand White rabbits or pigmented rabbits (Charles River Laboratories, http://www.criver.com, or Harlan Winkelmann, http://www.harlan.com); there were no systematic differences between strains, and the data were pooled. All procedures were approved by the animal care committee. After dark adaptation for two or more hours, rabbits were deeply anesthetized by intramuscular injection of ketamine (50 mg/kg body weight; Curamed Pharma/Pharmaselect, http://www.plasmaselect.com) and xylazine (Rompun, 10 mg/kg body weight; Bayer, http://www.bayer.com) and then sacrificed with intravenous pentobarbital (Narcoren, 160 mg/kg body weight; Merial, http://www.merial.com). The eyes were quickly enucleated, and the retinae were dissected in Ames medium under dim red illumination.

As described previously [35], a piece of mid-peripheral retina was flat-mounted in a the recording chamber [15,71] and superfused (at ≈2–4 ml/min) with warm (≈36 °C), oxygenated (95% O_2_, 5% CO_2_) Ames medium. A fluorescent dye (3 to 7 μM Sulforhodamine 101) was added to the bath, which allowed the location, size, and shape of retinal neurons to be visualized using two-photon fluorescence microscopy (2PM, see below and [72]). ON SACs were identified by their characteristic round 10 μm–diameter somata (Figure 1A). The inner limiting membrane (ILM) next to a targeted SAC was opened by scratching a hole in it with a patch pipette and expanding it by “blowing” Ames into the space under the ILM while viewing the retina by infrared video imaging with LED illumination. Cellular debris was cleared away using a suction pipette. All subsequent electrical and optical recordings were conducted in sulforhodamine-free medium.

Antagonists were added to Ames solution without ionic substitution at the following concentrations: 25–50 μM Gbz (GABAzine or SR-95531), 300 μM PTX (picrotoxin), 50–75 μM TPMPA (1,2,5,6-tetrahydro-pyridine-4-yl-methylphosphinic acid), 1 μM strychnine, 5–90 μM CdCl_2_, 70–100 nM ω-Conotoxin M7C. All chemicals were purchased from Sigma-Aldrich (http://www.sigmaaldrich.com) unless noted otherwise.

### Electrical recordings.

SACs were recorded using the whole-cell tight-seal (patch-clamp) technique [73] with 3–12 MΩ patch pipettes pulled from borosilicate glass (O.D.: 1.0 mm, I.D.: 0.58 mm, with filament; Hilgenberg, http://www.hilgenberg-gmbh.de) using a multi-clamp amplifier (Axon Instruments, http://www.axon.com). Data were acquired with a digitizer interface (Digidata 1322A) using pClamp 8 software (both, Axon Instruments) and analyzed off-line with IgorPro (Wavemetrics, http://www.wavemetrics.com). For current-clamp recordings, patch pipettes were filled with solution that contained (in mM): K-aspartate (or K-gluconate) 110–120, KCl, 5–10, NaCl 5, HEPES 5–10, MgCl_2_ 0–1, Mg-ATP 1, Na_2_-GTP 0.1–1. For voltage-clamp experiments, K-aspartate (or K-gluconate) and KCl in the filling solution were replaced by equal amounts of Cs-methanesulfonate and CsCl, respectively. In some experiments, 0.2–0.5 mM CaCl_2_, 2–5 mM HEDTA (N-(2-hydroxyethyl)ethylenediamine-N,N′,N′-triacetic acid) and NMG (4-N-methyl-D-glucamine) were added to the intracellular solution without discernable effect on the results. All filling solutions contained the fluorescent Ca^2+^ indicator (100–200 μM) Oregon Green 488 BAPTA-1 (OGB-1; Invitrogen, http://www.invitrogen.com). For current-clamp recording, the membrane voltage (*V*_m_) was low-pass filtered at 2 kHz and digitized at 5–10 kHz. In voltage-clamp mode, the current (*I*_m_) was digitized at 2 to 25 kHz and low-pass filtered at 1 kHz. Voltages (*V*_m_ and *V*_COM_) were corrected off-line by subtracting 15 mV for the liquid junction potential (calculated: 14 to 16 mV, measured: 17 to 18 mV; see [74]. Voltage-clamp recordings were, in addition, compensated off-line for series resistance (23 to 75 MΩ) effects. The average resting potentials were −62 ± 1 mV (*n* = 58; range −26 to −82 mV) and −48 ± 1 mV (*n* = 25; range −35 to −58 mV) with the K^+^- and Cs^+^- based filling solutions, respectively. The average input resistance (only determined for Cs^+^) was 164 ± 21 MΩ (*n* = 17; range 83 to 478 MΩ).

### Two-photon microscopy.

Two different custom-built upright 2PMs (both in-house designs), each with two detector channels, were used to image sulforhodamine fluorescence as well as to record Ca^2+^ indicator signals (Figure 1, and [15,75]). One microscope was equipped with a 20× water immersion lens (0.95 W, XLUMPlanFI, Olympus, http://www.olympus.co.jp/en/), to allow through-the-objective (TTO; see below) visual stimulation over a sufficiently large field. Different parts of the dendrite were imaged by shifting the laser-scanner offset without moving the objective lens, thus, leaving the TTO stimulus pattern stationary. The other microscope was equipped with through-the-condenser (TTC; see below) stimulation and a 60× water immersion lens (0.9NA; Leica, http://www.leica-microsystems.com). In both cases, the two-photon excitation source was a mode-locked Ti/sapphire laser (Mira-900; Coherent, http://www.coherent.com) tuned to approximately 930-nm wavelength. The scanning laser beam caused only moderate and/or quickly adapting electrical responses, which made it possible to record calcium signals in response to visual stimuli [75]. Spectrally nonoverlapping filters in the stimulation light path (see Light stimulation, below) and in front of the two detectors (“green channel,” Ca^2+^ indicator signal: D 535 BP 50 or 520 BP 30; “red channel,” Sulforhodamine: HQ 622 BP 36, Chroma/AHF, http://www.chroma.com), ensured that the 578- or 600-nm stimulus light did not interfere with the detection of fluorescence.

For Ca^2+^ imaging, SACs were filled with OGB-1 by diffusion from the patch pipette. The imaging software (CfNT) was written by R. Stepnoski (Bell Labs) and M. Müller (Max-Planck Institute for Medical Research). Small-image series (64 × 8 pixels at 62.5 Hz) were acquired from short dendritic segments and analyzed off-line with IgorPro (Wavemetrics). The observed changes in fluorescence suggest changes in [Ca^2+^] of hundreds of nM; no attempt was made at absolute quantification.

### Light stimulation.

Light stimuli were generated using custom-written Windows software compiled with Delphi7 (Borland, http://www.borland.com). Two different stimulus projection systems were used: In the TTC setup, the light stimuli were output via a video projector (Astrobeam 530, 80 Hz frame rate; 800 × 600 pixels; A+K, http://www.anders-kern.de), band pass-filtered (600 BP 20), and focused through the substage condenser (0.32 NA; Carl Zeiss, http://www.zeiss.com) onto the photoreceptors (≈4.4 μm/pixel; approximately 130–2,000 photons·s^−1^·μm^−2^ for 600 nm). In the TTO setup, stimuli were displayed using a miniature liquid crystal on silicon (LCoS) display (i-visor DH-4400VP; Cybermind Interactive, http://www.cybermindnl.com, or i-glasses VGA; EST, http://www.est-kl.com, 30 or 60 Hz frame rate; 800 × 600 pixels) illuminated by a band pass-filtered (578 BP 10) yellow LED. The output from the stimulator passed through some scaling and focus-correction optics as well as scan and tube lenses to be finally projected by the objective lens (≈1-mm field of view) onto the retina (stimulus resolution on the retina ≈ 2.1 μm/pixel; approx. 10,000–40,000 photons·s^−1^·μm^−2^ for 578 nm; measured with an Optical Power Meter, Model 840; Newport, http://www.newport.com). The focus-correction optics provides a shift, by up to approximately 100 μm, between microscope and stimulus focus planes. The stimulus thus remained focused on the photoreceptors while imaging dendrites in the inner plexiform layer.

With either stimulus projection system, stimulus contrast *(C)* was calculated using

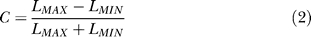
with *L*_MIN_ and *L*_MAX_ being the minimal and maximal light intensities, respectively, which were chosen to always be in the linear range of the display device. The velocities of the moving stimuli (gratings, circular waves) ranged from 0.5 to 2 mm/s (temporal frequencies: 2 to 10.5 Hz).

Circular wave stimuli were centered on the cell and consisted of expanding or contracting concentric sinusoidal waves (first term in Equation 3) with one full cycle per stimulus radius (*r*_Max_). Pixel values *(P)* were calculated using

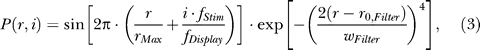
with the distance from the stimulus center (soma), *r* (in μm; 0 ≤ *r* ≤ *r*_Max_), the stimulus frequency *f*_Stim_ , the display refresh frequency *f*_Display_, and the frame index *i*. To avoid sharp edges and to restrict the motion to roughly the outer two-thirds of the SAC dendrites, the resulting sine wave was filtered with a “doughnut-shaped” super-Gauss (second term) with *r*_0,Filter_ = 0.5·*r*_Max_, and *w*_Filter_ = 0.562·*r*_Max_. To get the desired contrast, the values were then scaled by

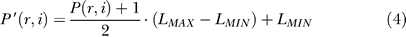
the background was kept at average intensity ((*L*_MAX_ − *L*_MIN_)/2), such that the stimulus consisted of alternating brighter- and darker-than-average waves (e.g., drawing in Figure 1D).

For most SACs recorded (dendritic field diameters ≈ 280 to 360 μm), we used *r*_Max_ = 192 μm, which means that the annular area with the circular wave extended between approximately 45 and 147 μm from the center. For the few cells that had smaller or larger dendritic fields, we used an *r*_Max_ of 128 or 256 μm, respectively. For the data shown in Figure 8A–8D, *r*_Max_ was 192 μm and the stimulus was presented only inside a broad ring (inner-outer diameter: Figure 8A: 90–360 μm; 8B: 90–270 μm; 8C: 90–210 μm; and 8D: 210–360 μm). The background was set to the mean intensity of the circular wave.

### Data analysis.

Fluorescence *(F)* data were spatially averaged over regions of interest (ROI) that covered a 5–15 μm–long section of a distal dendrite and temporally filtered (box-car filter with a 42-ms window) and background corrected using a ROI placed next to the dendrite. For each response, Δ*F*(t)/*F*_0_ was calculated (*F*_0_ being the prestimulus *F* averaged over 200 to 500 ms). For quantification, the area under the trace (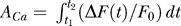 ) for a defined time interval (*t*_1_ < *t* < *t*_2_) was calculated (for details, see [15]).

For the Ca^2+^-activation curves (Figure 7), we normalized *A*_Ca_ for each cell to the Ca^2+^ response at −35 mV taken from the interpolated [Ca^2+^] versus *V*_COM_ relationship of the cell (with spline-interpolated Ca^2+^ values between voltage steps). The data points pooled from all cells were then fitted using (adapted from Equation 1 in [76]):

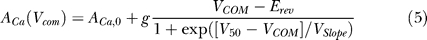
with somatic clamp potential *V*_COM_ and free parameters *A*_Ca,0_ (offset), *g* (max. conductance), *V*_50_ (half-activation voltage), and *V*_Slope_ (the slope voltage).

To quantify the electrical response to circular wave stimuli, we used spectral analysis by Fourier decomposition (Figure 2) of trace segments that contained an integer number of stimulus cycles and excluded the onset of the motion response (Figure 2A, black rectangle). Discrete Fourier decomposition can introduce spurious harmonic frequencies if the signals contain components (such as a stimulus-onset transient) that are not periodic with the stimulus frequency. Therefore, the analysis window was shifted in 50-ms steps along the trace until the residual (difference between data trace and reconstruction, see below) was minimal. Four components at different frequencies were extracted from this analysis: the DC component *V*_0_ (at 0 Hz), the fundamental, *V*_1_ (at *f*_1_), and the second and third harmonics, *V*_2_ (at 2·*f*_1_) and *V*_3_ (at 3·*f*_1_) (*I*_0_, *I*_1_, *I*_2_, and *I*_3_ for voltage-clamp measurements). These four components (each comprising amplitudes and, except for *V*_0_ or *I*_0_ phases) are sufficient to capture the response characteristics (Figure 2C, inset). To ensure reliable analysis, we only included cells with a robust light-evoked voltage response (≥2 mV) to CF motion of the standard circular-wave stimulus at the lowest used stimulus contrast ≥45%. To facilitate the comparison between cells, we used normalized asymmetry indices (*AI*_0..3_):

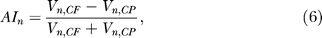
with *n* = 1, 2, or 3. A positive index means that CF motion is preferred. Note that, because only amplitude but no phase information is used, all indices equal zero does not imply identical waveforms. Although the absolute phases depend on the origin of the time window, the quantities 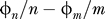 (relative phase) do not and thus can be used to characterize the waveforms (Figures 2C and 3C). The maximal response excursions were determined from a histogram of the voltages within the analysis window (*V*_Valley_ = 5%; *V*_Peak_ = 95%).

All averages are given as mean ± standard error of the mean (SEM). Statistical significance of differences was evaluated using the Wilcoxon matched-pair signed-ranks test [77] implemented in Matlab (Mathworks, http://www.mathworks.com) with *p* ≤ 0.05 considered as significant.

### Numerical evaluation of the two-compartment model.

The circuit diagram of the two-compartment model is shown in Figure 9B. Both compartments contain the same type of active conductance, which is modeled as a Hodgkin-Huxley–type channel, containing one *m* and one *h* element (e.g., [48]). For the *m* element, the activation probability follows

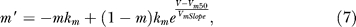
where *m′* denotes the time derivative of *m* and *k*_m_ the off-rate. Only the on-rate is assumed to be voltage *(V)* dependent, whereby *V*_m50_ and *V*_mSlope_ are the half-activation and slope voltages, respectively. The equation for the *h* element is identical. The voltages in the two compartments (*V*_P_ and *V*_D_) can be calculated by solving the following system of coupled differential equations:

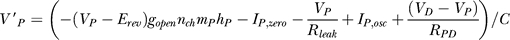


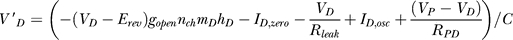


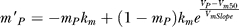


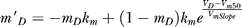


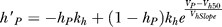


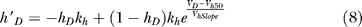



With longitudinal resistor (*R*_PD_), membrane capacitor *(C),* leak resistor (*R*_leak_), injected input currents (*I*_P,zero_, *I*_D,zero_, *I*_P,osc_, and *I*_D,osc_), and for the VGCs: maximal conductance (*g*_open_·*n*_ch_), reversal potential (*E*_rev_). For simulation parameters and results, see Figure 9 and Table 2.

## Supporting Information

Figure S1 Velocity Dependence (A) Voltage responses evoked by circular-wave stimuli with three different velocities (period: 192 μm; averages of 3 trials; CF motion in orange, CP in blue; *V*_Rest_ = −41 mV).
(B) Amplitudes of DC (*V*_0_), fundamental (*V*_1_), and harmonics *V*_2_ and *V*_3_ as a function of stimulus velocity (averages of five cells).
(487 KB TIF)

Figure S2 Holding-Voltage Dependence (Single-Cell Data) Amplitudes of the fundamental (*I*_1_), the second and the third harmonics (*I*_2_, and *I*_3_) as function of somatic clamp potential (*V*_COM_, as in Figure 5C) of the four cells (A–D) used for the calculation of the asymmetry indices (*AI*s) depicted in Figure 5D and three other cells (E–G) that showed only small second harmonics, close to the no-motion level, and were thus not included in the averaged *AI*s.
(870 KB TIF)

Protocol S1 Estimating the VGCC Slope Conductance and the Number of Ca^2+^ Channels Needed to Generate the Second Harmonic Signals (117 KB DOC)

## Abbreviations

AI: asymmetry index
CF: centrifugal
CICR: calcium-induced calcium release
CP: centripetal
DC: direct current
DS: direction selectivity, direction-selective
DSGC: direction-selective ganglion cell
HVA: high-voltage–activated
*I*_1_: current component at the fundamental frequency
*I*_2_: second harmonic current component
*I*_3_: third harmonic current component
LVA: low-voltage–activated
SAC: starburst amacrine cell
*V*_0_: direct current voltage component
*V*_1_: voltage component at the fundamental frequency
*V*_2_: second harmonic voltage component
*V*_3_: third harmonic voltage component
VGC: voltage-gated channel
VGCC: voltage-gated Ca^2+^ channel
