# Supplementary material for: A Dendrite-Autonomous Mechanism for Direction Selectivity in Retinal Starburst Amacrine Cells
Source: PLoS Biol. 2007 Jul 10;5(7):e185. doi: 10.1371/journal.pbio.0050185 (PMC1906843; doi:10.1371/journal.pbio.0050185)
Supplement: Supplementary file 1 [file pbio.0050185.sd001.doc]

PROTOCOL S1

Estimating the size of the slope conductance provided by the VGCCs present in SACs.

We first assume for the channel open probability (Eqn. 2.22 in [48]):

,	(S1)

where $V_{50}$ is the voltage at which half the channels are open and $V_{slope}$ is the activation slope voltage. The total current is:

$I\left(V\right)=n_{ch}g_{0}p_{open}\left(V\right)\left(V-E_{rev}\right)=n_{ch}I_{sc}\left(V\right)$,	(S2)

with the number of channels *n_ch_*, the open-channel conductance $g_{0}$(which can be assumed to be constant, however, only for voltages sufficiently far from $E_{rev}$), the reversal potential *E_rev_*, and the single channel current$I_{sc}$. For the slope conductance ($\alpha=\partial I/\partial V)$ per channel we get

.	(S3)

For *E_rev_* = 46.8 mV, $V_{50}$ = -10.9 mV, $g_{0}$= 3 pS , and $V_{slope}=4.56$ mV (*g*_0_ from [58], other numbers derived from fits to P/Q-type Ca^2+^-channel data from [45], see legend of Fig. 7E), $\alpha\left(V\right)$ can become as negative as -8.22 pS (at ‑12.2 mV), which for ≈ 930 channels, as are needed to account for *I*_2_ (see below), amounts to a negative slope conductance $n_{ch}\alpha=-930\cdot8.22pS$ ≈ 1/(‑130 MΩ).

Estimating the number of Ca^2+^ channels (*n_ch_*) needed to generate the 2nd harmonic signals.

Since we are interested in moderate voltage modulation amplitudes (*V_A_*) we can expand the I-V curve into a Taylor series about *V*_0_,

$\begin{matrix} I\left(V-V_{0}\right)=I_{0}+n_{ch}\left[\left.\frac{\partial I_{sc}\left(V\right)}{\partial V}\right|_{V=V_{0}}\left(V-V_{0}\right)+\left.\frac{1}{2}\frac{\partial^{2}I_{sc}\left(V\right)}{\partial V^{2}}\right|_{V=V_{0}}\left(V-V_{0}\right)^{2}+O\left(V-V_{0}\right)^{3}\right]= \\ =I_{0}+n_{ch}\left[\alpha\left(V-V_{0}\right)+\beta\left(V-V_{0}\right)^{2}+O\left(V-V_{0}\right)^{3}\right] \end{matrix}$ , (S4)

where $I_{0}$is the current when $V=V_{0}$. With sinusoidal voltage modulation, $V-V_{0}=V_{A}\cos\left(\omega t\right)$, with $V_{A}$ the modulation amplitude, the current modulation is

, (S5)

where "..." indicates frequency components at 3ω or higher. Solving for the number of channels we find:

.	(S6)

Using the I-V curve for P/Q-type channels (with $E_{rev}$=  46.8 mV, $V_{50}$ = -10.9 mV, $g_{0}$= 3 pS, and $V_{slope}=4.56$mV) we find$\beta$= 687 pA/V^2^ by calculating the magnitude of the second derivative (in the curve for P/Q-type in Fig. 7E this is at ‑18 mV, where the curvature becomes maximally negative) using Eqn. S2. If we neglect inactivation and kinetic aspects and use *V_A_* = 5 mV (Fig. 1D) with the channel parameters listed above, we find that ≈ 930 channels are needed to get *I_2_* = 8 pA (Fig. 5C).
